# DEATH NARRATIVE IN 19TH-CENTURY CHINA: HOW DID NEWSPAPERS FRAME DEATH AND DYING?

**DOI:** 10.1093/geroni/igz038.1021

**Published:** 2019-11-08

**Authors:** Yifan Lou

**Affiliations:** Columbia University, New York, New York, United States

## Abstract

This study explored the death narrative in the late Qin dynasty as expressed in Chinese newspapers in the 19th century. Using textual analysis to analyze the 646 pieces of news containing death-related topics, this study revealed the discourse regarding death and dying during this period can be understood at three levels: (a) euphemism of death: the language of death and its relationship with power and social hierarchy; (b)definition of “good death”: including preferences for location, cause, and experiences of death and dying; and (c) Western influence on the death narrative: missionaries’ efforts to incorporate Catholic and Chinese traditions to attract more believers. This paper argues that the current Chinese people’s perception of death is inherited and evolved from those historical roots, which has practical implications for the systematic development of hospice care in China. Suggestions include changing the language used in the hospice policy, emphasizing the importance of confidentiality in home-based hospice programs, and building a hospice system based on public perceptions of so-called “good death” while advocating for individualized definitions of this concept.

